# Silk Film Topography Directs Collective Epithelial Cell Migration

**DOI:** 10.1371/journal.pone.0050190

**Published:** 2012-11-21

**Authors:** Brian D. Lawrence, Zhi Pan, Mark I. Rosenblatt

**Affiliations:** 1 Department of Biomedical Engineering, Cornell University, Ithaca, New York, United States of America; 2 Department of Ophthalmology, Weill Cornell Medical College, New York, New York, United States of America; Université de Technologie de Compiègne, France

## Abstract

The following study provides new insight into how surface topography dictates directed collective epithelial cell sheet growth through the guidance of individual cell movement. Collective cell behavior of migrating human corneal limbal-epithelial cell sheets were studied on highly biocompatible flat and micro-patterned silk film surfaces. The silk film edge topography guided the migratory direction of individual cells making up the collective epithelial sheet, which resulted in a 75% increase in total culture elongation. This was due to a 3-fold decrease in cell sheet migration rate efficiency for movement perpendicular to the topography edge. Individual cell migration direction is preferred in the parallel approach to the edge topography where localization of cytoskeletal proteins to the topography’s edge region is reduced, which results in the directed growth of the collective epithelial sheet. Findings indicate customized biomaterial surfaces may be created to direct both the migration rate and direction of tissue epithelialization.

## Introduction

Basement membranes are sheet-like extracellular matrix (ECM) structures located on the basal side of polarized cells, and provide a complex nanoscale topography that offers physical support and chemical ligand binding sites for cell attachment and migration [1]–[3]. In addition, basement membranes are broadly involved in many physiological processes such as tissue repair, embryogenesis, and morphogenesis [2], [4]–[10]. The interaction of basement membrane surface topography with surrounding cells may profoundly influence cell functions through a phenomenon known as contact guidance. The phenomenon of contact guidance is characterized by the response of cells to structures on the nano to micro scale and result in changes in local cell adhesion, polarization, migration, and differentiation [11]–[15]. The topography of the human corneal epithelial basement membrane has been characterized to be a felt-like arrangement of fibers with processes and pores that have feature sizes in the nano- and microscale range [5], [16]. Further work has demonstrated that the presence of the surface topography is important for corneal epithelial adhesion, migration, and tissue development [13], [15], [15], [17], [17]–[20].

The effects of contact guidance are mainly observed as an alignment of the cytoskeleton, elongation of cell morphology, and oriented cell migration in the direction of the underlying patterns [20]–[23]. Such studies have shown that the topographic cues, independent of biochemistry and mechanics, can significantly influence cell behaviors. Additionally, data suggests that the size and shape of patterned substrates can direct the differentiation of progenitor cells [5], [24]. These investigations provide evidence that the topographic cues may not only regulate cell phenotypic behaviors, but also profoundly influence gene expression. As a result the naturally occurring cues to illicit contact guidance could play an important role in regulating cell function during various biological processes. Investigation is currently underway to apply such biomimetic principles into the development of tissue-engineered constructs, including corneal tissue-engineering [5], [19], [25], [26].

Prior studies of contact guidance have primarily focused on the behavior of single cell suspensions cultured on silicon wafers or synthetic polymer surfaces. However, the use of silicone wafers or non-biocompatible materials do not integrate with the surrounding tissue environment and make *in vivo* translation problematic. Furthermore, seeding of single cells for the study of epithelial contact guidance does not recapitulate the *in situ* environment where epithelium spreads over surfaces *en masse* [1], [3], [27]. During corneal homeostasis and wound healing, corneal epithelial cells are not isolated, but instead proliferate and migrate as collective sheets with a myriad of intercellular interactions that may be greatly influenced by the presence of topographic features. As a result such studies may not be optimal from both a physiological and *in vivo* translation standpoint. The current study circumvents these issues by investigating the influence of contact guidance on more physiologically relevant cell sheets cultured on a highly biocompatible silk film substrate.

Developing biomaterial technology is a foundational area of research in the emerging fields of tissue-engineering and regenerative medicine. Regenerated silk fibroin protein solution is considered to be a novel choice of material selection due to the protein’s inherent biocompatibility and nonimmunogenic properties when implanted *in vivo* [28]–[30]. Fibroin protein offers a number of advantages over other biopolymers due to the controllable nature of their material properties, which is derived from induced changes within the protein’s secondary structure (i.e. beta-sheet and alpha helix structures) [31], [32]. By modifying the silk fibroin secondary structure content fundamental material properties such as degradation, mechanical strength, and geometric structure can be defined at a greater extent then other biologically derived molecules like collagen, chitosan, alginate, and polyester based polymers [28], [33]. In addition, silk films cast from fibroin protein solution are transparent and possess tunable material properties in terms of mechanical strength, degradation rate, geometric design, and both surface chemistry and topography [2], [4], [11], [34]. These properties combined with inherent biocompatibility and transparency make it uniquely suited for use in the cornea as a clinical tool and for tissue-engineering purposes [2], [5], [6], [8], [9], [35].

It has also been shown that silk films can be produced with nanoscale surface topographies at a resolution higher than what can be achieved by other comparable biopolymers such as collagen, alginate, and hyaluronic acid [11]–[15], [26], [36]–[38]. This is largely due to the fact that silk fibroin begins as an amorphous polymer and is not fibrillar like collagen, and that water-annealed silk films have limited swelling when in the presence of water which does not distort patterned feature topography like most other hydrophilic biopolymers [39]. This makes this material optimally suited for assessing topographic effects on cell and tissue growth [5], [16], [40], [41]. In addition, due to silk fibroin’s high surface patterning resolution capabilities it allows researchers to look at features only once accessible through the use of synthetic, and largely non-biocompatible, surfaces [42].

The current study aims to better understand how surface topography affects epithelial cell sheet migration, which is an essential property for successful healing of the ocular surface after injury [13], [15], [17], [20], [43]. Here, silk films with a patterned microsurface topography were seeded with human corneal-limbal epithelial (HCLE) cells to study the effect of these surface structures on corneal epithelial sheet migration. Findings from this study will contribute to the knowledge of how collective corneal epithelial cell sheets are influenced by topographic cues in their microenvironments and how these interactions influence cell behaviors. Findings will support ongoing efforts to better understand how fundamental surface design criteria may be produced to enhance clinical applications in ocular surface repair and cornea tissue engineering.

## Materials and Methods

### Production of Silk Solution

*Bombyx mori* silkworm cocoons (Tajima Shoji Co., Yokohama, Japan) were cut into thirds and then boiled for 40-minutes in 0.02 M Na_2_CO_3_ (Sigma-Aldrich) to extract the glue-like sericin proteins from the structural fibroin proteins as previously described [5]. The solution was dialyzed in water for 48-hours (MWCO 3,500, Pierce, Inc.). The dialyzed silk solution was centrifuged twice at 13,000-g, and the supernatant collected and stored at 4°C. The final concentration of aqueous silk solution was 8 wt./vol.%.

### Production of Patterned Silicon Wafer Surfaces

Silicon wafer features were designed to have a 2-µm width, 4-µm pitch, and 1.5-µm depth using L-edit software (Tanner EDA, Inc.) and then imported into a DWL66 laser pattern generator and direct write machine (Heidelberg Instruments, Heidelberg, Germany). The finished mask was then placed within an Autostep 200 DSW i-line wafer stepper (GCA, Inc.) using 100-mm diameter silicon wafers coated with 1-µm thick layer of photoresist (Megaposit™ SPR™ 220-3.0, Dow Chemical, Inc.). A 21-die array was created in a [3∶5∶5∶5∶3] design with 10-mm diameters separated by 5-mm spacing. The wafers were then placed into a Unaxis 770 ion etching device (Plasma-Therm, LLC, St. Petersburg, FL).

### PDMS Casting Surface Preparation

Flat poly dimethylsiloxane (PDMS) substrates were produced by pouring 5-mL of solution (Momentive, Inc., Albany, NY) onto the patterned silicon wafer surfaces. The cast PDMS solution was then degassed for 2-hours under vacuum, and then cured in an oven at 60°C overnight. The following day the cured PDMS was removed from the silicon substrate and then punched to form round 14-mm circles centered on the patterned regions.

### Silk Film Casting and Sterilization

Water insoluble silk films 40-µms in thickness were cast using 75-uL of 8% silk fibroin solution cast upon the round PDMS surfaces as previously described [5]. Silk film samples were removed from their PDMS surfaces, and sterilized in 70% EtOH for 15-minutes and washed with PBS. Silk film samples and glass control surfaces were then placed pattern side up into 24-well plates, and a stainless steel O-ring (15.4-mm OD, 11.6-mm ID, Superior Washer, Inc., Hauppauge, NY) was placed on top to hold the film down to the culture well bottom surface.

### Cell Culture and Collective Cell Migration Assay

A previously established immortalized human corneal-limbal epithelium (HCLE) cell line was kindly provided by Dr. Ilene Gipson (Harvard Medical School, Boston, MA) [27]. All cell media supplies were purchased from Invitrogen (Eugene, OR). The cells were cultured in keratinocyte serum free medium (K-SFM, Invitrogen) with 1% 100× penicillin-streptomycin, 0.3 M CaCl_2_, 0.45 vol.% bovine pituitary extract (BPE), and 0.2 ng/mL of epithelial growth factor (EGF, Human recombinant). In order to create a collective cell sheet, 1-uL droplet of cell suspension was seeded upon the silk films and glass cover slip controls for 30-minutes before adding media.

### Cell Droplet Dispersion Assay

Cell droplets seeded upon the various surfaces were incubated for 1-day. Phase contrast images were taken to record the shape of the collective cell sheets (n = 4) using a 10×objective lens (NA 0.45 air, Carl Zeiss, AG). A mosaic of several images were combined together using the MozaiX program within the Zeiss AxioVision software package in order to view the entire culture. The aspect ratio of the seeded culture was the measured.

### CyQuant Nucleic Acid Content Assay for Cell Proliferation

The CyQuant NF (Invitrogen, Inc., Eugene, OR) assay was used after 1 and 5 days of cell culture by adding 500-uL of CyQuant NF solution to each sample (n = 3 for each surface) for 2-hours at 37°C. Sample fluorescence intensity was then measured using a SpectraMax M2 fluorimeter microplate reader (Molecular Devices, Inc., Sunnyvale, CA) with excitation wavelength at 485-nm and emission wavelength collected at 530-nm.

### Collective Cell Migration Assay and Analysis

Collective cell sheet migration was monitored 1-day post-seeding upon the silk film and glass surfaces. Cell migration was monitored using the microscope’s 24-well plate micro-incubator (PeCon, GmbH; M24 S1). Time-lapse phase contrast imaging was utilized to record a frame every 10-minutes over a 10-hour period. A subset of confluent HCLE cultures were incubated for 2-hours in media containing 4-ug/ml of the mitotic inhibitor Miomycin-C (MMC, Sigma-Aldrich). Another subset of samples received mouse anti-E cadherin (AEC) at a concentration of 5-ug/ml (SHE78-7, Invitrogen). As a comparative measure for leader cell formation upon the various surfaces tortuosity values (τ) for the cell sheet border was assessed by measuring the arc-chord ratio from the extracted images of the time-lapse videos, which is defined as:
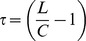
Where L is the total curve length of the migrating cell sheet border, and C is the measured linear distance between the ends curve length ends as measured using the ImageJ (ver. 1.45, NIH).

Cell sheet migration rate was quantified by exporting phase contrast images from the time-lapse movies at initial 0-hour and 10-hour time points in culture. Quantification was accomplished by using the ‘Distance between Polylines’ plugin to measure the average distance and respective standard deviation between cell sheet borders (n = 4). Single cell migration was analyzed using the ‘Tracking’ package in AxioVision software for individual time-lapse movies. Randomly sampled cells from various cell sheet cultures (n = 20, N = 4) were tracked from representative locations of the cell sheet on silk and glass surfaces. The software compiled measurements for total distance, straight distance, tortuosity, and migration rate. In addition, the cell sheet migration efficiency (η) was calculated as:
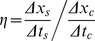
where 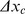 and 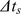 is the change in cell sheet movement and corresponding times respectively, while 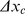 and 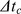 is the change in single cell movement and corresponding times respectively. Standard deviation were taken as the quotient propagated error.

### Immunofluorescent Staining and Imaging

Samples were fixed with 4% paraformaldehyde in PBS for 15-minutes, permeabilized with 0.4% Triton in PBS for 5-minutes, and blocked with 2% BSA in PBS for 30-minutes at room temperature. Focal adhesions and GTPase proteins were visualized by immunostaining with a 1∶600 dilution of primary antibodies for vinculin (V9131, Sigma, St. Louis, MO), RhoA (ab54835, Abcam, Cambridge, MA), phosphorylated RhoA (p.RhoA, ab41435, Abcam, Cambridge, MA), and Rac1+Cdc42 (ab18758, Abcam, Cambridge, MA) for 1-hour at room temperature, followed by incubation with Oregon Green 488 goat anti-mouse secondary antibody (O11033, Invitrogen, Eugene, OR) at a 1∶800 dilution for 1-hour. F-actin and nuclei were then stained by incubating cells in 1∶100 dilution of Alexa Fluor 568 Phalloidin (A12380, Invitrogen, Inc., Eugene, OR) and 1∶10,000 dilution of DAPI (83210, AnaSpec, San Jose, CA) for 20 and 5-minutes respectively, while protected from light. Samples were placed on a glass slide and covered with a glass cover slip and mounting media (Vectashield, Vector Laboratories, Inc., Burlingame, CA). Fluorescent images were taken using 63×objective lens (NA 1.4 oil, Carl Zeiss, AG). The number of vinculin-positive focal adhesions was quantified by using ImageJ, and a linescan of actin fibers was obtained by using MetaMorph software (Molecular Devices, Inc., Sunnyvale, CA).

### Western Blot Analysis of GTPase Protein Content

HCLE cell sheets were cultured as described above upon the various silk film surfaces and glass substrates for 2-days. The cell media was removed, and each sample was washed 2× with PBS at 4°C. Following PBS removal, a 200-uL sample of RIPA buffer with protease and phosphatase inhibitors (100× Halt™ single-use cocktail, Thermo Scientific, Rockford, IL) was added to each sample to release intracellular protein. The cell protein was collected using a 1-cm cell scraper device (BD Biosciences, Bedford, MA), and the cell lysate solution was then collected and added to the next sample well to increase the concentration of protein. The resulting protein lysate was mixed on ice for 15-minutes and then centrifuged at 14,000-RCF for 15-minutes (5415 D, Eppendorf, Inc.).

The protein concentration of each sample was determined using the bicinchroninic acid (BCA) assay (Thermo Scientific, Rockford, IL) as previously described [44]. Next, NuPAGE® LDS Sample Buffer (4×, Invitrogen, Inc.) and protein ladder (10747-012, Invitrogen, Inc.) were added to each sample. Samples were heated at 70°C for 10-minutes. The NuPAGE® gel system (1.0 mm×15-well, 4-12% Bis-Tris, Invitrogen, Inc.) was used with 1×NuPAGE® MES SDS Running Buffer (20×, Invitrogen, Inc.) on a constant 200-V setting for 40-minutes. Proteins were transferred using the iBlot® Gel Transfer Device (Invitrogen, Inc.). The membrane was placed into infrared (IR) compatible blocking buffer (Odyssey®, LI-COR Biosciences) for 1-hour. Next, 1∶600 primary antibody dilutions of RhoA (ab54835, Abcam, Cambridge, MA), phosphorylated RhoA (p.RhoA, ab41435, Abcam, Cambridge, MA), and Rac1+Cdc42 (ab18758, Abcam, Cambridge, MA) were incubated with the membrane at 4°C for 24-hours and then washed 3×with 0.1% TWEEN® 20 (Sigma-Aldrich, Inc.). The appropriate infrared dye labeled secondary antibody solutions were prepared at a dilution of 1∶2,000 (IRDye®, LI-COR Biosciences, Inc.) for vinculin, GAPDH, and actin in blocking buffer, and membranes were incubated for 1-hour covered from light. Membranes were analyzed using an infrared Odyssey machine (LI-COR Biosciences, Inc.). Protein densitometry measurements were then attained and ratios from the various sample readings were calculated.

## Results

### Silk Film Surface Topography Affects Cell Sheet Migration

**Figure 1:**
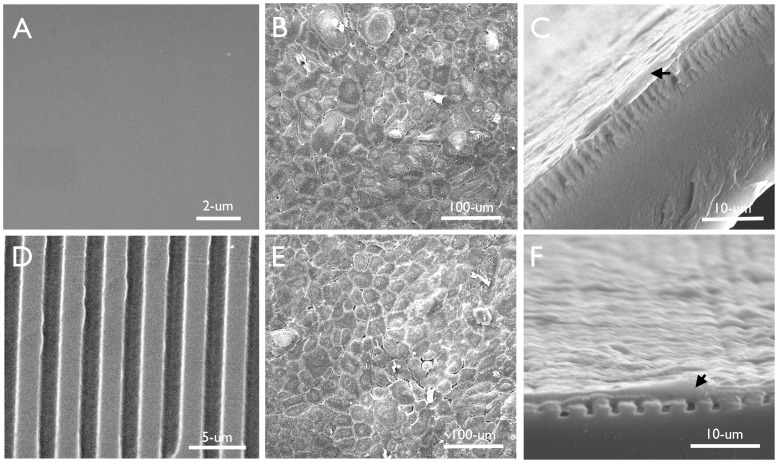
HCLE cells cultured on silk film substrates. (A) Flat silk film surfaces were absent of significant surface roughness, and (B) sustained healthy cell sheet cultures with (C) apparent cellular adherence to the culture surface. (D) Desired lined patterns of 4-µm feature pitch dimensions with 2-µm ridge widths were formed upon the silk film surfaces, and (E) sustained healthy cell sheet cultures with (F) apparent cellular adherence to the top surfaces of the patterned surface topography.

Micro-patterned silk films were successfully cast from PDMS molds with both flat and patterned surfaces. SEM imaging revealed that flat silk film surfaces were free from significant roughness [Figure 1A], and that patterned feature dimensions were produced for surfaces with parallel line surface features [Figure 1B]. In addition, the desired feature depth of 1.5-µm along with consistent feature wall formation was also attained as illustrated by cross sectional imaging analysis [Figure 1F]. Cultured cell sheets appeared to demonstrate healthy and confluent morphologies at 1-day in culture upon both flat [Figure 1B] and patterned [Figure 1E] surfaces. Additional cross-sectional analysis indicated that cells formed adherent contact with both flat [Figure 1C] and patterned silk culture substrates [Figure 1F]. Cells adhered to the apical surface of the patterned silk films and did not contact the entire feature depth as desired.

**Figure 2:**
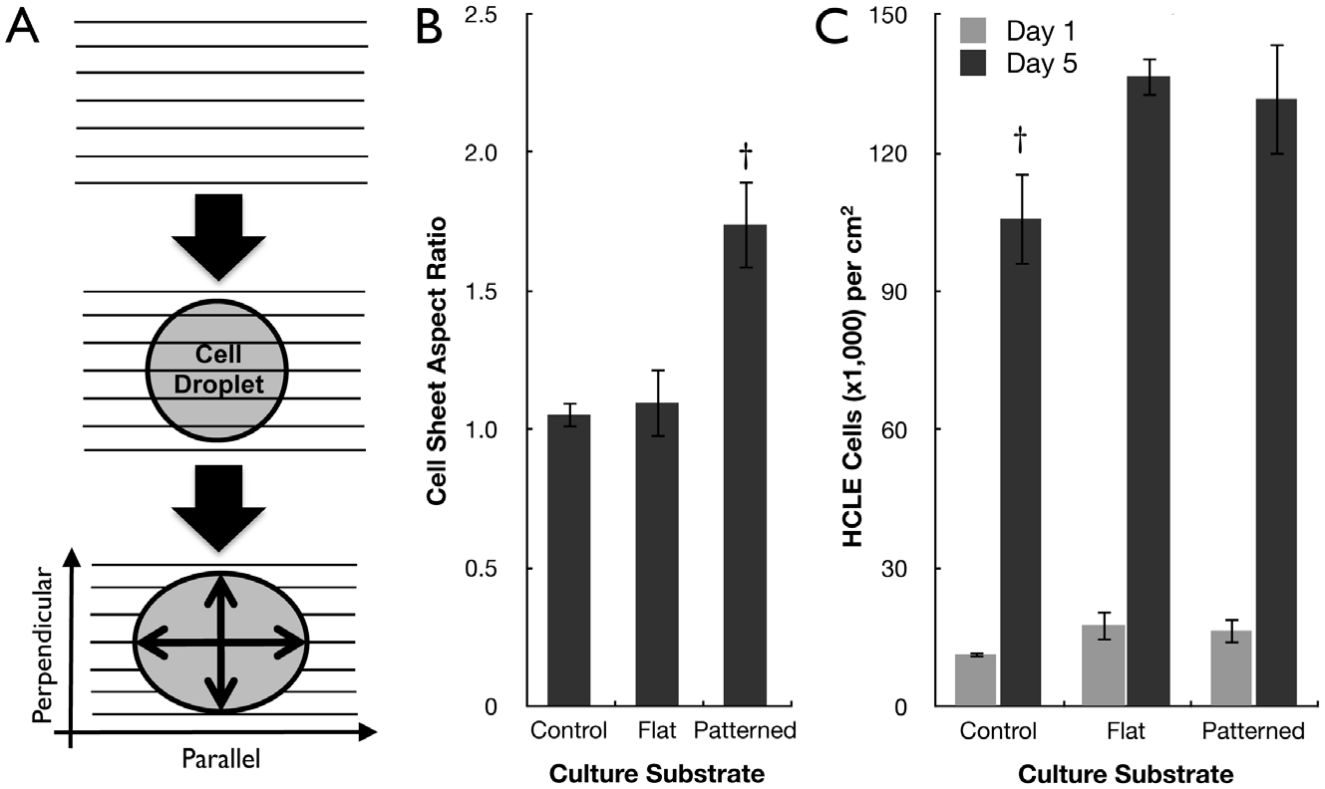
Cell sheet growth directed by silk topography. (A) Schematic demonstration of a cell droplet forming a collective sheet that disperses parallel to the patterned silk feature axis as measured by the culture length to width aspect ratio, with parallel versus perpendicular directions defined. (B) The aspect ratios of cell sheets on patterned silk surfaces after 1-day in culture (n = 4, †indicates p<0.05 when compared other substrates, error bars = SD). (C) Cell proliferation on various surfaces at 1-day and 5-days in culture as quantified by nucleic acid content (n = 3, †indicates p<0.05 when compared to other substrates of similar group, error bars = SD).

High-density cell suspension droplets were successfully seeded upon the silk film surfaces and glass controls within 2-hours post-seeding. Whole cell sheet dispersion was then characterized upon the various surfaces by measuring the length and width dimensions of each droplet over time [Figure 2A]. After 1-day in culture the measured cell sheet aspect ratio upon patterned silk film surfaces was increased by 60% on average (p<0.05) when compared to flat silk surfaces and glass controls [Figure 2B]. Analysis indicated cell sheets dispersed in a parallel direction along the silk film’s patterned feature edge. However, on flat silk and glass surfaces the cell sheets dispersed in a uniform isotropic pattern. Nucleic acid content was assessed to confirm that cell sheet dispersion differences were not primarily a function of culture proliferation. Cell proliferation upon glass controls showed no significant difference when compared to silk surfaces after 1-day in culture, while both flat and patterned silk surfaces demonstrated a significant increase in cell proliferation compared to glass controls after 5-days in culture [Figure 2C]. The results indicate that cell sheets have a greater propensity to proliferate on silk film surfaces when compared to glass controls over time. However, no proliferative difference existed between the flat and patterned silk surfaces indicating that cell sheet dispersion was not a function of cell proliferation.

**Figure 3:**
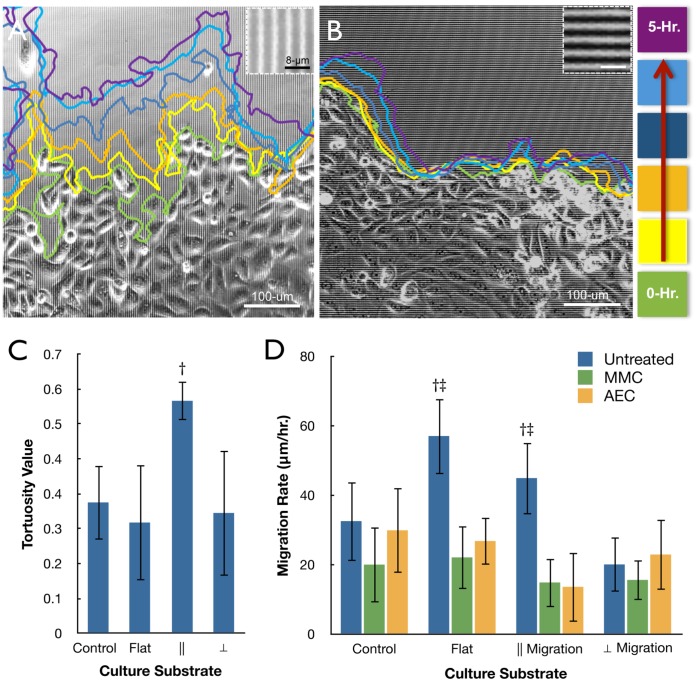
Cell sheet migration on silk film culture surfaces. Phase contrast images of migrating cell sheets on patterned silk films substrates traveling in the (A) parallel or (B) perpendicular direction of the feature edge as indicated by the inset images of the patterned surfaces, and color tracings are taken at 1-hour increments as indicated by the colored legend. (C) Tortuosity measurements for the migrating cell sheet border on glass controls, flat silk, and patterned silk surfaces accounting for sheet movement parallel (||) and perpendicular (⊥) to the feature edge axis respectively. (D) Cell sheet migration rate on glass controls, flat silk, and || or ⊥ directions to the patterned silk surfaces for untreated, MMC treated, and AEC treated cultures (n = 4, †‡indicate p<0.05 to the patterned ⊥ direction within groups and between treatment groups respectively, error bars = SD).

A dynamic study of collective cell migration was performed using phase-contrast time-lapse imaging of the epithelial cell sheets. Based on the dispersion trends witnessed between flat [Video S1] and patterned surfaces [Video S2-S3] attention was focused on the cell sheet boundary regions. For patterned surfaces with lined features this regions was separated into cells migrating perpendicular [Video S2] or parallel [Video S3] to the silk film feature edge axis. Flat surfaces were measured in accordance to the major cell sheet migratory axis. When comparing patterned silk film surfaces, cell sheet migration rate was greater for movement parallel to the patterned feature edge [Figure 3A], while limited dispersion occurred perpendicular to the patterned edge direction [Figure 3B]. A detailed observation showed a greater presence of leader cell activity ahead of the migrating cell sheet for cultures moving parallel to the patterned feature axis, while appearing absent for sheets migrating in the perpendicular direction. This was quantified by measuring tortuosity (τ) for the cell sheet migration border regions. Analysis revealed that τ had a significant 2-fold increase (n = 4, p<0.05) for cells migrating in the parallel direction on patterned surfaces when compared to perpendicularly migrating cell sheets and flat surfaces [Figure 3C]. The value for τ was not significantly different between the other culture surfaces.

Migration rate was assessed over a 10-hour period by comparing cell sheet borders at both 0 and 10-hour time points. The distance traveled between these two time points was quantified upon silk culture surfaces and glass controls to determine the affect of surface topography on migration rate. In addition, cell sheets were cultured in the presence of the mitotic inhibitor Mitomycin-C (MMC) or anti-E-cadherin (AEC) antibodies to evaluate the effect of cell proliferation and cell-to-cell interaction respectively. It was found that untreated cells had the highest rate of migration on flat silk surfaces, which was almost twice as fast on average when compared to glass control surfaces (p<0.05, n = 4) [Figure 3D]. In addition, a significant over 2-fold drop in migration rate was found on flat surfaces treated with MMC and AEC antibodies respectively (p<0.05, n = 4). In addition, a 2-fold drop in migration rate was also observed on patterned silk film surfaces treated with MMC and AEC respectively (p<0.05, n = 8).

Sheet migration rate in a direction parallel or perpendicular to the patterned feature edge orientation was also assessed on pattered silk film substrates. It was found that cell sheet movement parallel to the feature edge direction was over 2-fold higher on average (p<0.05, n = 4) when compared to cell sheet movement perpendicular to the feature edge direction for untreated cultures [Figure 3D]. In addition, a drop in migration rate was shown to exist for cells moving in the parallel direction when cultures were treated with either MMC or AEC respectively. No change in migration rate was observed for cultures migrating perpendicular to the feature edge for either treatment.

### Topographic Influence on Individual Cell Alignment and Migration

**Figure 4:**
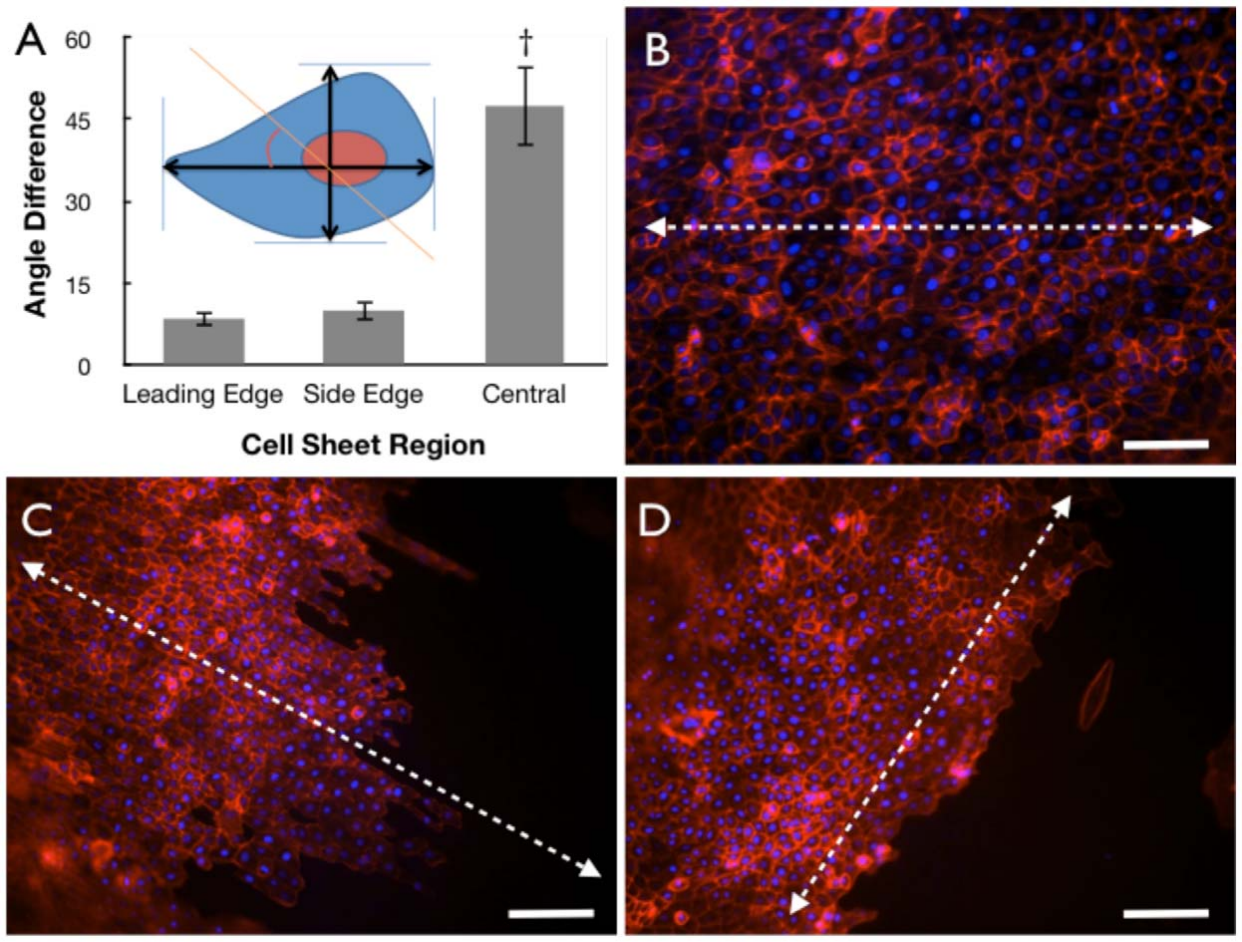
Border cells oriented by silk substrate topography. (A) Polarity of HCLE cells at the leading edge, side edge, and central regions of the cell sheet, with inset schematic illustrating measurement method (n = 4, †indicates p<0.05, error bars = standard deviations). (B–D) Actin (red) and nuclei (blue) staining of cells located within the (B) central region and (C–D) border regions of the migrating cell sheet with arrows indicating patterned feature edge axis (scale bar = 100-µm).

The polarity of individual cells upon each surface was characterized through actin staining of the cell sheet cultures upon patterned silk surfaces. The deflected angles from the major length of the cell to the surface pattern feature edge axis were quantified respectively [Figure 4A]. Imaging revealed that the cell polarity was increased at the boundary edges as indicated by a reduction in the measurement of the deviation of cell major axis alignment with the surface pattern axis. Near the boundary edge of the cell droplet this deviation measured approximately 10°, while within the center region of the droplet this deviation was increased to 45° degrees [Figure 4A]. Thus, cells within the center region of the droplet possessed a random orientation with respect to the patterned feature axis [Figure 4B], while cell orientation demonstrated a polarity along the patterned feature edge at sheet boundary regions [Figure 4C–D]. The individual cell polarity at the sheet edges correlated with the direction of cell migration observed from time-lapse experiments. Cell sheet migration preferred movement in the parallel direction of the patterned feature axis [Figure 4C]. Correspondingly, cell migration was less preferred for cell sheet migration perpendicular to the patterned feature axis [Figure 4D].

**Figure 5:**
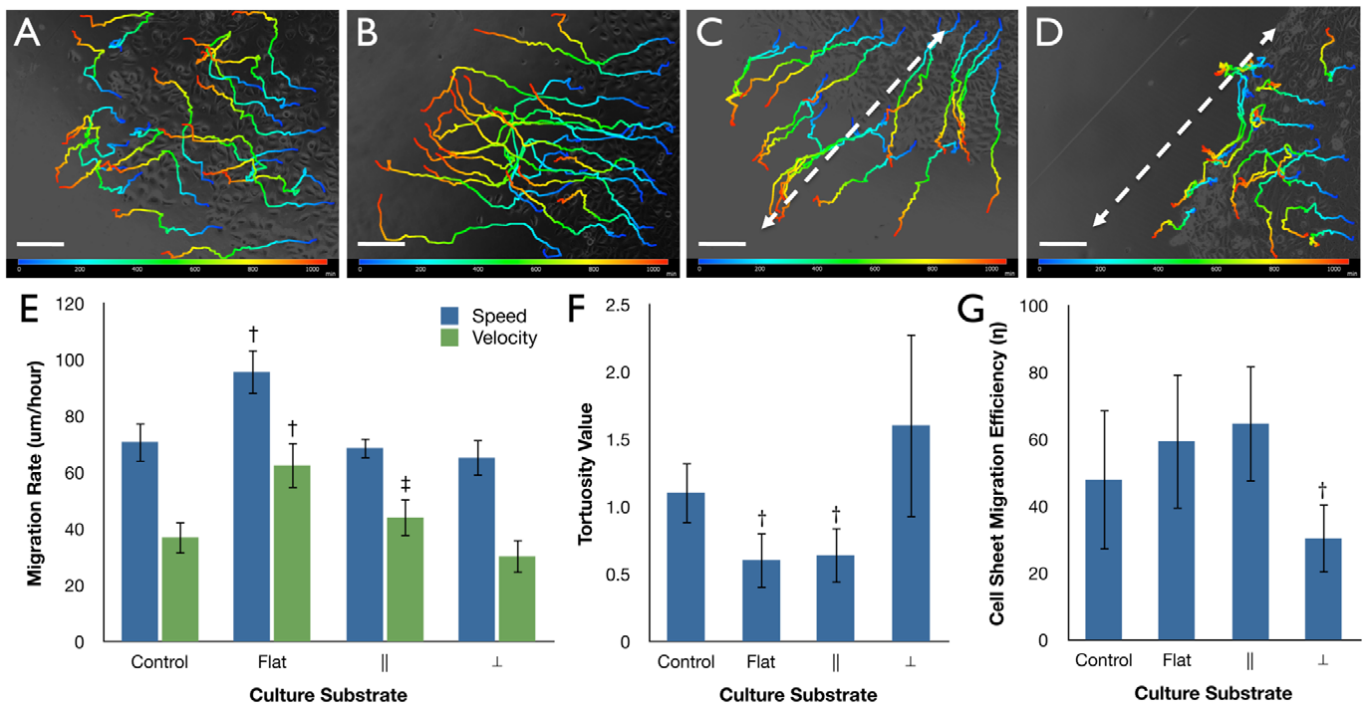
Individual cell migration path on silk substrates. Migratory paths of individual HCLE cells on (A) glass control, (B) flat silk, (C) parallel (||) and (D) perpendicular (⊥) movement to the patterned silk topography over a 10-hour culture period (N = 4, n = 20). Color indicates time 0 (blue) to 10-hour (red) time points, and white dashed arrows indicate direction of the silk topography (scale bars = 200-µm). Singular cell migratory measurements for (E) total distance and straight distance measurements (†indicates p<0.05 when compared to culture substrates in similar groups, ‡indicates p<0.05 when compared to ⊥), (F) tortuosity values (†indicates p<0.05 when compared to control and ⊥ substrates), and (G) migration rate for singular cellular movement upon various substrates (†indicates p<0.05 when compared to all other substrates). (H) Cell sheet migration efficiency (η) for cultures migrating on the various substrates conditions (†indicates p<0.05 when compared to all other substrates). Error bars = SD for all graphs.

Migratory paths of individual HCLE cells within the previously observed migrating cell sheets were analyzed from the time-lapse videos for the various surfaces [Figure 5A–D]. In addition, single cells migrating on patterned silk films in both parallel and perpendicular directions to the feature edge were also studied. Initial qualitative assessment indicated that cells migrating on flat surfaces and in the parallel direction of the feature edge appeared to move radially away from the cell origin point, while perpendicularly traveling cell trajectories tended to follow the feature edge. Further migratory analysis revealed that total distance and straight distance traveled for single cells was significantly higher (N = 4, n = 20, p<0.05) on flat silk film surfaces when compared to all other culture substrates [Figure 5E]. However, it was also shown that straight distance traveled was significantly higher for cells moving in the parallel direction to the feature edge when compared to cells moving in the perpendicular direction (N = 4, n = 20, p<0.05). Tortuosity measurements were significantly lower (N = 4, n = 20, p<0.05) for single cells migrating on flat and in the parallel direction of patterned silk films when compared to glass controls, and nearly 3-fold higher on average than perpendicularly migrating cells [Figure 5F]. Migration rate was also significantly higher for single cells on flat silk film surfaces when compared to other substrates (N = 4, n = 20, p<0.05) (Figure 5G). When comparing cell sheet migration efficiency (η), which is measured as the cell sheet migration velocity over the respective individual cell migration velocity, it was observed that η for perpendicularly moving cultures was nearly 3-fold less than cells traveling either on flat silk surfaces or parallel to the topography, and η was approximately 2-fold less than glass control surfaces [Figure 5G].

### The Effect of Silk Film Surface Patterning on Cytoskeleton Formation

**Figure 6:**
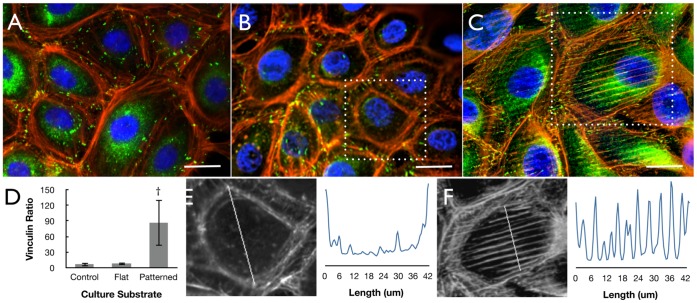
Cytoskeletal alignment to silk substrate topography. Fluorescent microscopy images of HCLE cells on (A) glass, (B) flat silk, and (C) patterned silk surfaces (nuclei - blue, actin – red, and vinculin - green, scale bars = 20-µm). (D) Quantification of intracellular to cellular periphery FA formations as indicated by vinculin staining. Fluorescent images and corresponding line-scan analysis of actin fiber distribution of HCLEs cultured on (E) flat and (F) patterned silk film surfaces, where the inset white line of each image indicates region of analysis.

In order to better understand how cell-surface interactions influence collective cell migration, further investigation into the distribution of actin and vinculin protein formation was assessed. Initial observations revealed that cell cultures on both glass [Figure 6A] and flat silk film surfaces [Figure 6B] had actin fibril and focal adhesion (FA) formations along the basal cell body periphery. Patterned silk film surfaces appeared to induce alignment of actin fibrils and FA formation within the central region of the cell basal surface localized along the feature edges [Figure 6C]. Aligned features present in cells were located at both the cell sheet border region and internally in the cell sheet. Analysis of vinculin formation upon the various surfaces revealed an over 10-fold increase on average in FA localization within the central basal area of cells cultured upon patterned surfaces when compared to cultures on flat surfaces [Figure 6D]. Actin fibril alignment on the silk film surfaces were further investigated using line-scan analysis of single HCLEs within the cell sheet culture. It was observed that actin fibril alignment was not present on flat silk film surfaces [Figure 6E], while directed fibril alignment was observed on the various patterned surfaces [Figure 6F]. The absence of actin fibril formation within the intracellular region was visualized as a U-shape pixel intensity profile after performing a line-scan analysis, while periodic peaks were seen on patterned silk surfaces corresponding with the presence of the feature edges.

**Figure 7:**
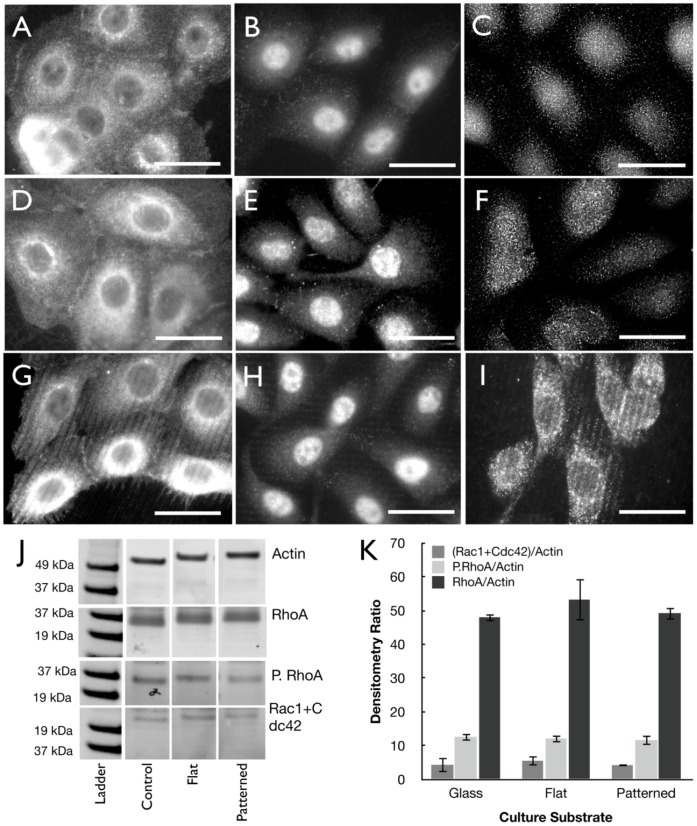
GTPase localization directed by silk substrate topography. Immunofluorescent imaging of RhoA, P.RhoA, and Rac1+Cdc42 antibody binding for (A–C) glass, (D–F) flat silk, and (G–H) patterned silk surfaces respectively. (J) Western blot membrane staining and (K) densitometry results for the various GTPases (n = 3, error bars = SD).

To better understand the mechanistic basis of guided actin fibril alignment along the patterned silk film feature edge GTPase protein localization was visualized using immunofluorescent microscopy on HCLEs grown on flat and pattern silk films and glass control substrates. Imaging revealed that RhoA, phosphorylated RhoA (P.RhoA), Rac1 and Cdc42 proteins were distributed throughout the cytoplasm for cultures on glass control surfaces [Figure 7A–C] and flat silk film surfaces [Figure 7D–F]. However, on patterned silk films surfaces GTPase proteins were found to concentrate along the feature edge axis [Figure 7G–I]. Western blot analysis was undertaken to assess GTPase content for cultures grown on the various surfaces. Results indicated that RhoA, P.RhoA, Rac1, and Cdc42 proteins were isolated in similar quantities from the various culture substrates [Figure 7J–K]], and indicates that the patterned surfaces induce a localizing effect.

## Discussion

For the first time patterned silk film surfaces were successfully used to evaluate collective corneal epithelial cell migration *in vitro*. This work demonstrates that the presence of edge topography plays a significant role in directing entire cell culture movement through what appears to be an inhibitory effect on individual cell movement. Previous work studying the effects of surface topography on cell migration has primarily focused on signle cell migration. A variety of patterned biopolymer surfaces like collagen, hyaluronic acid, and poly-lactic acid do in indeed appear to direct cell migration [26], [45], [46]. However, limited studies have observed collective cell migration on patterned surface topographies, which is typically observed on non-patterned substrates [1], [47], [48]. Recent work has shown that the geometry of a given surface topography may play a role in directing kidney cell grouping and growth characteristics in culture [49]. However, overall culture migration was not assessed. The current study builds off the previous findings in the literature by combining both a patterned silk biomaterial substrate with a collective cell migration culture model.

The above results indicate that silk film surface topography may be utilized to influence whole cell sheet properties, such as culture migration rate and direction. Therefore, by designing a surface that is specified to influence individual cell movement entire culture growth may be directed. In addition, due to the transparency and accessibility of the silk film culture system [40], these effects were observed over multiple dimensional magnitudes from the macroscopic shaping of the cell sheet down to the level of subcellular protein localization. The silk film micro-topography was shown to affect overall cell sheet growth. Whole cultures aligned along the feature edge axis on patterned silk surfaces, while growing isotropically on flat silk and glass control surfaces. Such macroscopic effects must be derived from a more fundamental process at the individual cellular level, which was further explored.

The micro-to-macro effects of epithelial cell sheet directional alignment appeared to be accomplished through changes in migration rate as dictated by cellular orientation to the patterned feature edge. Collective sheets migrating parallel to the topography edge axis move at a rate 2-3 fold faster than cells moving in a perpendicular approach. Similar results have been shown for NIH 3T3 cells as well in which migratory direction is dictated by the presence of the surface topography, and that feature topography aspect ratios influence migration speed significantly [50]. Upon the silk film patterns, cells located at the cell sheet border tend to elongate parallel to the feature edge, which aids to accelerate migration when compared to regions of the sheet moving in the perpendicular approach. However, cells located centrally within the sheet present limited elongation and are less affected by the presence of the topography. Therefore, the topography’s edge acts to inhibit collective migration by acting on individual cells located at the border regions and moving in a perpendicular approach [12], [51].

The overall effect that each individual cell has on moving the collective sheet is significantly decreased for migration perpendicular to the edge topography. The edge acts similar to a guiding track for individual cell migration for parallel movement. Previous work has demonstrated that fibroblast cell orientation in culture can be readily directed using patterned silk film surfaces [41], [50]. This guiding track effect on individual cells is amplified at the level of the collective cell sheet where the *en masse* movement is slowed in the perpendicular direction of the feature edge. In more descriptive terms, cell migration efficiency (η) can be taken to be the cell sheet migration rate, or useful energy output, over the average individual cell migration rate, or total energy input, for the collective sheet. Thus, η in a given direction can be controlled through the use of edge topography by modulating the degree of uniformity for directed individual cell movement.

**Figure 8:**
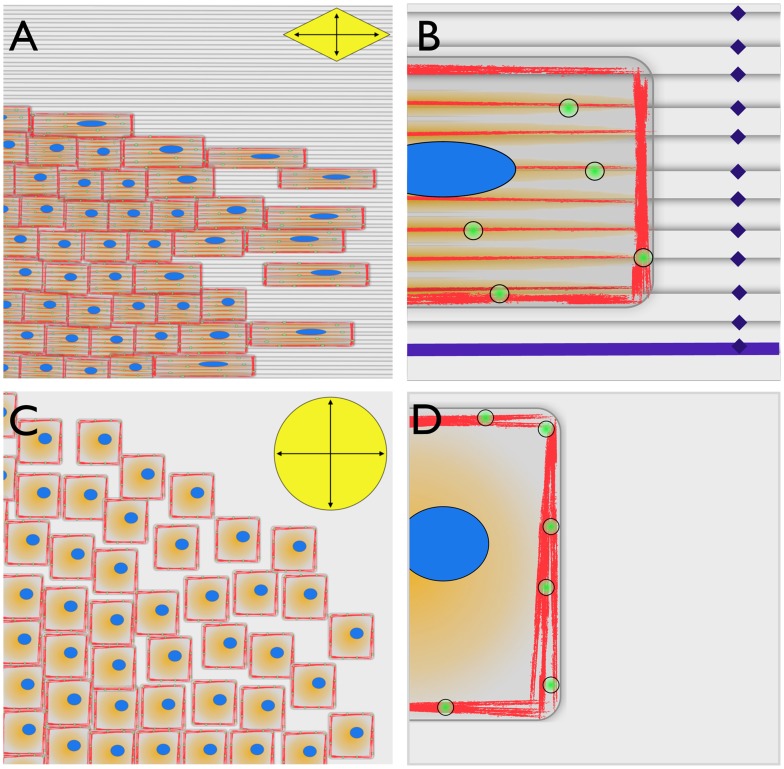
Schematic representations of cell sheet migration. Representative renditions of cell sheet migration on (A-B) patterned and (C-D) flat silk film surfaces. (A) Cell sheet migration on patterned silk surfaces was compact in nature with leading border cells elongating in the parallel direction of the patterned feature edge. Migration rate was 2-fold higher in the parallel direction of the feature edge when compared to perpendicular movement direction as indicated by the vectors contained in yellow diamond. (B) Actin fibril (red), FA (green), and GTPase (orange) proteins were found to preferentially localize along the surface feature edges as indicated in purple coloring. (C) In contrast, cell sheet migration on flat silk film substrates was less compact with leading cells maintain a normal cuboidal morphology. Migration rate was similar in all directions as indicated by the vectors contained in the yellow circle. (D) Actin fibrils (red) and FAs (green) were found to primarily localize at the cellular basal periphery region with GTPase proteins (orange) localizing uniformly within the central region of the cell.

In regions where edge topography is located there was an increased preference for actin fibril and FA formation. The observed localization of protein at the feature edge surface has been previously observed in other studies and is attributed to a contact guidance effect from the surface topography [14], [24]. Here, this effect manifests itself at the level of the individual cells where movement is preferentially guided in the parallel direction of the topography edge. As a result η in a given direction is largely dictated by the collective sum of preferred individual cell movements. Individual cells prefer moving parallel to the edge topography where there is a reduction in the number of regions that enhance protein localization, and ultimately inhibits collective cell migration less. On the other hand cell sheets on flat silk surfaces appear to move in a less compacted and isotropic pattern upon the culture surface. These findings are supported by previous observations where the destination of individual cell movement is preferred for regions with higher regions of edge density (i.e. smaller feature sizes) [52]. The absence of surface features does not influence protein localization and as a result actin and FA formation appear to remain isolated to the cell periphery. Singular cells travel faster and less compactly due to the lack of directional guidance, which results in a uniform η in all directions. A summary illustration of these findings is shown in Figure 8.

### Conclusions

The results above demonstrate a new understanding into the role of contact guidance in directing individual cell behavior within a collective epithelial cell sheet to produce a concerted tissue-level response. Directed localization of cytoskeleton components occurs in response to the presence of silk film surface topography by contact guidance. The topography directs individual cell movement parallel to the feature’s edge axis, which creates a collective cell response resulting in the reduction of migration rate for epithelial sheets moving perpendicular to the feature edge axis. Individual cells appear to prefer traveling in directions where edge topography is less prevalent, which increases cell sheet migration efficiency. Future work may build off these results to design cell surfaces that may more effectively direct cell sheet migration, with the hope of utilizing such surfaces to enhance cell responses such as reepithelialization rate post trauma.

## Supporting Information

Video S1 **Time-lapse phase-contrast microscopy video of a representative HCLE cell sheet migrating on a flat silk film surface over a 10-hour period.**
(MP4)

Video S2 **Time-lapse phase-contrast microscopy video of a representative HCLE cell sheet migrating on a patterned silk film surface in the perpendicular direction of the topography edge axis over a 10-hour culture period.**
(MP4)

Video S3 **Time-lapse phase-contrast microscopy video of a representative HCLE cell sheet migrating on a patterned silk film surface in the parallel direction of the topography edge axis over a 10-hour culture period.**
(MP4)
